# Harmine targets inhibitor of DNA binding‐2 and activator protein‐1 to promote preosteoclast PDGF‐BB production

**DOI:** 10.1111/jcmm.16562

**Published:** 2021-05-07

**Authors:** Jie Huang, You‐You Li, Kun Xia, Yi‐Yi Wang, Chun‐Yuan Chen, Meng‐Lu Chen, Jia Cao, Zheng‐Zhao Liu, Zhen‐Xing Wang, Hao Yin, Xiong‐Ke Hu, Zheng‐Guang Wang, Yong Zhou, Hui Xie

**Affiliations:** ^1^ Department of Orthopaedics Xiangya Hospital Central South University Changsha China; ^2^ Movement System Injury and Repair Research Center Xiangya Hospital Central South University Changsha China; ^3^ Department of Sports Medicine Xiangya Hospital Central South University Changsha China; ^4^ National Clinical Research Center for Geriatric Disorders Xiangya Hospital Central South University Changsha China; ^5^ Department of Orthopaedics the Third Xiangya Hospital Central South University Changsha China; ^6^ Hunan Key Laboratory of Organ Injury, Aging and Regenerative Medicine Changsha China; ^7^ Hunan Key Laboratory of Bone Joint Degeneration and Injury Changsha China

**Keywords:** AP‐1, harmine, Id2, PDGF‐BB, preosteoclast

## Abstract

Osteoporosis is one of the most common metabolic bone diseases affecting millions of people. We previously found that harmine prevents bone loss in ovariectomized mice via increasing preosteoclast platelet‐derived growth factor‐BB (PDGF‐BB) production and type H vessel formation. However, the molecular mechanisms by which harmine promotes preosteoclast PDGF‐BB generation are still unclear. In this study, we revealed that inhibitor of DNA binding‐2 (Id2) and activator protein‐1 (AP‐1) were important factors implicated in harmine‐enhanced preosteoclast PDGF‐BB production. Exposure of RANKL‐induced Primary bone marrow macrophages (BMMs), isolated from tibiae and femora of mice, to harmine increased the protein levels of Id2 and AP‐1. Knockdown of Id2 by Id2‐siRNA reduced the number of preosteoclasts as well as secretion of PDGF‐BB in RANKL‐stimulated BMMs administrated with harmine. Inhibition of c‐Fos or c‐Jun (components of AP‐1) both reversed the stimulatory effect of harmine on preosteoclast PDGF‐BB production. Dual‐luciferase reporter assay analyses determined that PDGF‐BB was the direct target of AP‐1 which was up‐regulated by harmine treatment. In conclusion, our data demonstrated a novel mechanism involving in the production of PDGF‐BB increased by harmine, which may provide potential therapeutic targets for bone loss diseases.

## 1 INTRODUCTION

Blood vessels play a key role both in promoting bone growth and maintaining bone homeostasis via transporting oxygen, nutrients, minerals and metabolic waste.^1^, ^2^ In recent years, a specific vessel subtype named type H vessel (high expression of CD31 and endomucin) is identified in murine skeletal system.^3^, ^4^ Type H vessel couples angiogenesis and osteogenesis and its abundance is negatively correlated with ageing both in murine and human skeletal system.^3^, ^5^ A number of studies have shown that increasing type H vessel formation is capable of preventing bone loss and accelerating fracture healing.^6^, ^7^, ^8^ Preosteoclast, derived from myeloid precursor, is a mononuclear tartrate‐resistant acid phosphatase (TRAP)‐positive cell with abundant numbers on bone surface.^9^, ^10^ Our previous studies indicated that platelet‐derived growth factor‐BB (PDGF‐BB) secreted by preosteoclasts stimulates type H vessel and bone formation in ovariectomized mice,^11^ suggesting a new strategy to counteract osteoporosis by promoting preosteoclast PDGF‐BB‐induced type H vessel formation.

Harmine, originally isolated from seeds of peganum harmala, is a natural β‐carboline alkaloid which is widely distributed in the nature.^12^ Harmine possesses various biological properties including anti‐tumour, anti‐microbial, anti‐inflammatory and anti‐osteoclastogenesis effects.^12^, ^13^, ^14^ Our previous study has revealed that harmine is capable of preventing bone loss via increasing preosteoclast formation and PDGF‐BB production.^6^ However, the molecular mechanisms by which harmine facilitates PDGF‐BB production from preosteoclasts remain to be deciphered.

Inhibitor of DNA‐binding family (Id family: Id1‐Id4) is a member of helix‐loop‐helix (HLH) proteins, which can inhibit the differentiation of a number of cell lineages including osteoclast.^15^, ^16^ Previous study has shown that harmine is able to inhibit the fusion of preosteoclasts into osteoclasts by up‐regulating Id2.^13^ Thus, we supposed that Id2 might play a positive role in harmine‐enhanced PDGF‐BB production by increasing the numbers of preosteoclasts. Activator protein‐1 (AP‐1), a dimer composed of Fos (c‐Fos, FosB, Fra‐1 and Fra‐2) and Jun (c‐Jun, JunB and JunD) proteins, is a transcriptional complex critical for RANKL‐induced osteoclast differentiation of macrophages. It is unclear whether the AP‐1 transcription factor is involved in the harmine‐induced stimulation of preosteoclast formation and PDGF‐BB production in RANKL‐activated macrophages.

This study aimed to uncover the underlying molecular mechanism through which harmine promotes preosteoclast PDGF‐BB production.

## 2 METHODS

### 2.1 Cell cultures

Primary murine bone marrow macrophages (BMMs) were harvested from the tibiae and femora of 6 week‐old male mice. We flushed out bone marrow cells with injector and cultured the flushed cells in α‐MEM supplemented with 10% fetal bovine serum (FBS) (Gibco), 30 ng/mL recombinant murine macrophage colony‐stimulating factor (M‐CSF; PeproTech), 100 U/mL penicillin and 100 μg/mL streptomycin (Solarbio). 14 hours later, the adherent cells were discarded, the floating cells were collected and cultured in new flask for obtaining macrophages. For the osteoclastogenic culture, BMMs were administrated with 100 ng/mL receptor activator for nuclear factor κB ligand (RANKL; PeproTech) with or without harmine (3 μmol/L) for 7 days.

### 2.2 TRAP staining

We identified preosteoclast and osteoclast using a commercial TRAP kit (Sigma‐Aldrich). Briefly, the cells were fixed with 4% paraformaldehyde for 20 minutes. After being washed three times with PBS, cells were incubated with TRAP buffer according to the manufacturer's instructions. TRAP‐positive (red) mononuclear cells and multinucleated (three and more nuclei) cells were counted as preosteoclast and osteoclast, respectively.

### 2.3 Quantitative real‐time PCR (qRT‐PCR) analysis

Total RNA was isolated using TRIzol Reagent (Takara) according to the manufacturer's instructions. The concentration of RNA was determined by measuring the optical density at wavelengths of 260 nm and 280 nm. 1 μg of the total RNA was used to generate cDNA with the First Strand cDNA Synthesis kit (Bimake). Then, qPCR was performed using FastStart Universal SYBR Premix ExTaqTM II (Takara Biotechnology) on an ABI PRISM^®^ 7900HT System (Applied Biosystems). The relative gene expression was analysed by the relative standard curve method (2^−ΔΔCT^) with Gapdh as the reference. The primers used for qPCR are listed in Table S1.

### 2.4 Western blot

Total proteins were extracted from cells with RIPA lysis buffer containing protease inhibitor (Thermo Fisher Scientific). After quantifying their concentrations, samples were separated by sodium dodecyl sulphate‐polyacrylamide gel electrophoresis (SDS–PAGE) and transferred to polyvinylidene fluoride (PVDF) membranes (Millipore). After blocking with 5% non‐fat milk for 2 hours at room temperature, the membranes were incubated overnight at 4°C with the respective primary antibodies including c‐Fos antibody (1:200, Santa Cruz), c‐Jun antibody (1:200, Santa Cruz), Id2 antibody (1:200, Santa Cruz) and β‐Actin antibody (1:200, ProteinTech). Subsequently, the membranes were incubated for 1 hour with horseradish peroxidase (HRP)‐conjugated secondary antibodies (1:5000, ZenBio). The bands were visualized using an enhanced chemiluminescence (ECL) kit (Thermo Fisher Scientific). β‐Actin was used as an internal control.

### 2.5 Enzyme‐linked immunosorbent assay (ELISA)

The supernatant form BMMs was harvested and stored at –80°C until analysis. The concentration of PDGF‐BB was detected with commercial ELISA kit (Elabscience) following the instructions provided by the manufacturer.

### 2.6 Transfection of small interfering RNA (siRNA)

The siRNAs and transfection reagent were purchased from RIBOBIO (Guangzhou). The transient transfection was performed according to the manufacturer's instructions. Briefly, BMMs were seeded in 6‐ or 48‐well plates and transfected with 50 nmol/L Id2, c‐Fos, c‐Jun siRNA or control siRNA using transfection reagent. At 48 hours post‐transfection, the cultured media were exchanged for fresh media containing 100 ng/mL RANKL with or without harmine (3 μmol/L). After 7 days incubation, the cells and their supernatants were harvested for further experiments. The transfection efficiency was determined by qRT‐PCR and Western blot analysis.

### 2.7 Dual‐luciferase reporter assay

Luciferase reporter plasmids inserted promoter of PDGF‐BB containing the putative wild‐type (WT) or mutant (MUT) binding sites for AP‐1 were synthesized by Genscript Biotech Corporation. BMMs were seeded in 48‐well plates and transfected with the WT plasmid, MUT reporter plasmid or basic plasmid along with the internal control vector Renilla using Lipofectamine**^™^** 3000 (Invitrogen). After 48 hours of transfection, the cells were cultured in fresh media containing 100 ng/mL RANKL with or without harmine (3 μmol/L). The luciferase activity was determined by dual‐luciferase reporter gene assay system (Promega) and was normalized to Renilla luciferase activity following the manufacturer's instructions.

### 2.8 Statistical analysis

The quantitative data were shown as mean ± SD. Two‐group comparison was performed using unpaired, two tailed student's *t* test. One‐way analysis of variance (ANOVA) with Bonferroni post hoc test was employed for multiple‐group comparisons. *P* < .05 was considered statistically significant.

## 3 RESULTS

### 3.1 Harmine increases the number of preosteoclasts and the production of PDGF‐BB

**FIGURE 1:**
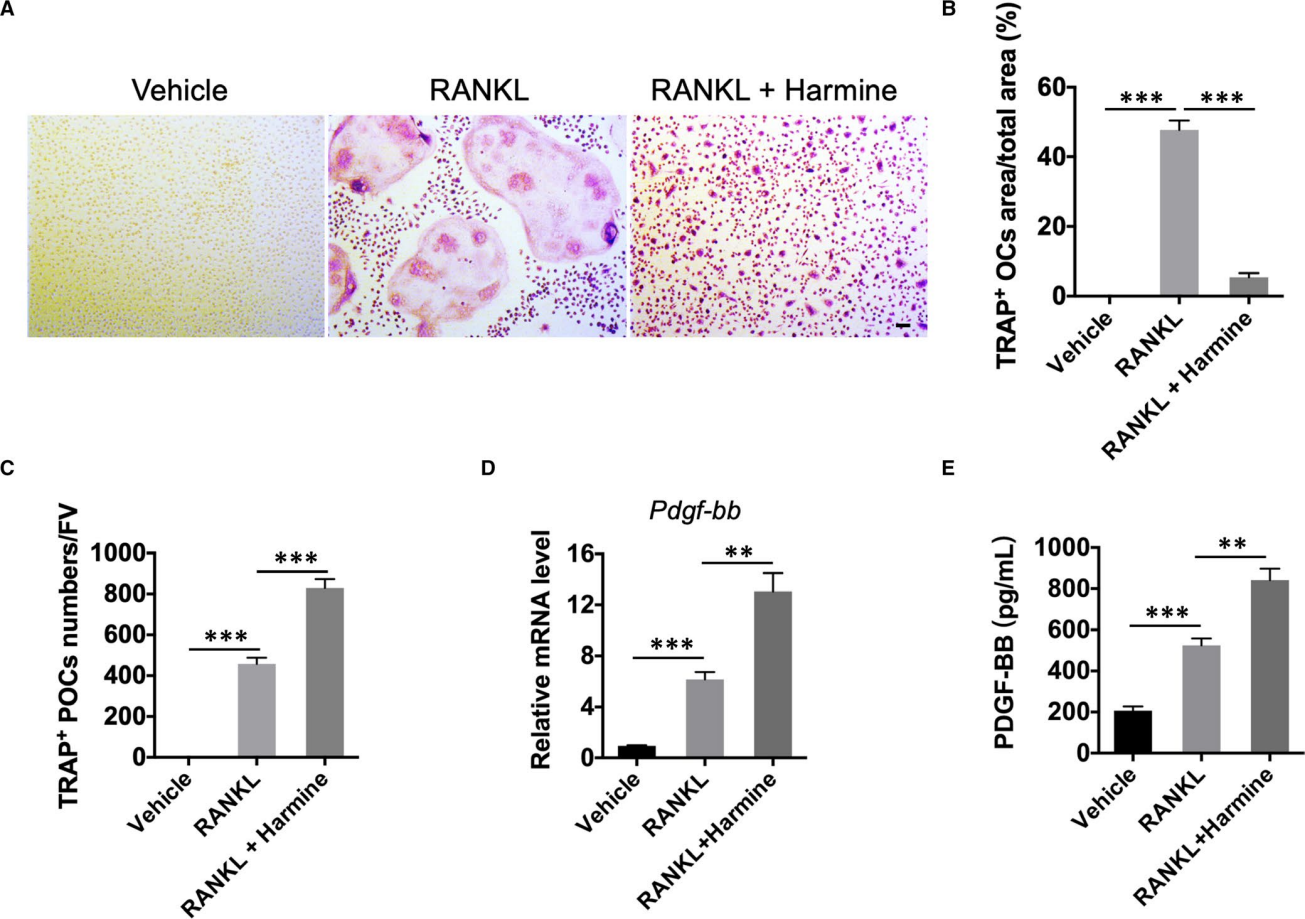
Harmine increases the number of preosteoclasts and the production of PDGF‐BB. A, Representative images of TRAP staining showing osteoclasts and preosteoclast formation from BMMs administrated with vehicle, RANKL, or RANKL + harmine. scale bar = 50 μm. B, Quantitative analysis of the area of TRAP‐positive osteoclasts (OCs). n = 3 per group. C, Quantification of the TRAP‐positive preosteoclasts (POCs). FV: field of view. n = 3 per group. D, qRT‐PCR analysis of PDGF‐BB mRNA expression levels. n = 3 per group. E, PDGF‐BB concentration in conditioned media from BMMs. n = 3 per group. *P < .05, **P < .01, ***P < .001

To detect the effects of harmine on preosteoclasts formation and PDGF‐BB production, we isolated BMMs from the tibiae and femurs of 6 week‐old C57BL/6 mice. The cells were induced by M‐CSF and RANKL with administration of harmine or vehicle (DMSO) for 7 days. Osteoclastogenesis was determined by TRAP staining. The results showed that most of the RANKL‐stimulated BMMs differentiated into TRAP‐positive mononuclear osteoclasts (preosteoclasts) and multinucleated osteoclasts, whereas harmine administration inhibited the fusion of mononuclear preosteoclasts into multinucleated osteoclasts, resulting in a significantly higher number of preosteoclasts relative to that of RANKL + vehicle treatment group (Figure 1A‐C). Then, we harvested the cells and their supernatant for evaluation of the production of PDGF‐BB. qRT‐PCR analysis demonstrated that RANKL treatment increased *Pdgf‐bb* mRNA levels compared to the un‐induced group, and harmine further enhanced the expression of *Pdgf‐bb* mRNA (Figure 1D). ELISA confirmed that the supernatants from BMMs treated with RANKL contained a higher concentration of PDBF‐BB protein compared to the un‐induced group, and the production of PDGF‐BB was further increased when BMMs were treated with harmine. In accordance with these results, in vivo studies also demonstrated that ovariectomized mice treated with harmine showed a markedly increased quantity of preosteoclasts, significantly higher levels of bone marrow PDGF‐BB and much more type H vessel in bone compared with those of vehicle group (Figure S1). To further confirm whether the effect of harmine on angiogenesis is mediated by preosteoclast‐derived PDGF‐BB, human microvascular endothelial cells (HMECs) were incubated with the conditioned media from BMMs stimulated by RANKL or RANKL + harmine for tube formation assays. As shown in Figure S2, the conditioned media from RANKL + harmine‐stimulated BMMs significantly facilitate tube formation ability of HMECs, and the positive effect was markedly abated by PDGF‐BB‐neutralizing antibodies. These observations suggest that harmine increases the formation of preosteoclasts, the production of PDGF‐BB, and thus enhances type H vessel formation.

### 3.2 Up‐regulation of Id2 partially contributes to the harmine‐induced promotion of preosteoclast formation and PDGF‐BB production

**FIGURE 2:**
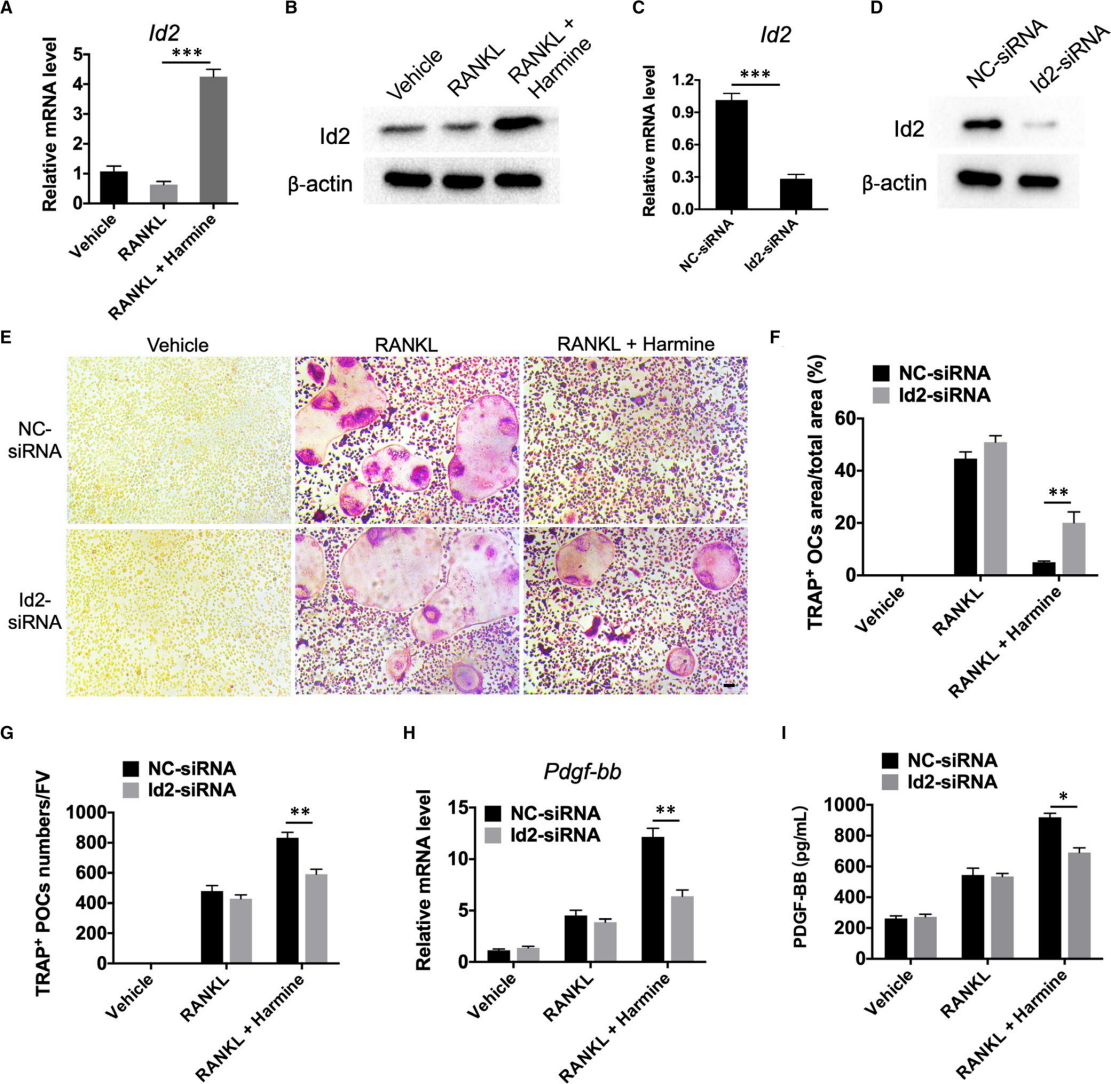
Harmine increases the formation of preosteoclasts via up‐regulation of Id2. A, B, Expression levels of Id2 in BMMs treated with vehicle, RANKL, or RANKL + harmine were assessed by qRT‐PCR A, and Western blotting analysis B, n = 3 per group. C, D, Transfection of BMMs with Id2‐siRNA led to a decrease in Id2 expression levels as verified by qRT‐PCR C, and Western blotting analysis D, n = 3 per group. E, Representative TRAP staining images showing osteoclast and preosteoclast formation from negative control (NC)/Id2‐siRNA‐transfected BMMs administrated with vehicle, RANKL, or RANKL + harmine. scale bar = 50 μm. F, Quantitative analysis of the area of osteoclasts in E, n = 3 per group. G, Quantification of the number of preosteoclasts in E, n = 3 per group. H, qRT‐PCR analysis of PDGF‐BB mRNA expression levels in NC/Id2‐siRNA‐transfected BMMs receiving different treatments. n = 3 per group. I, PDGF‐BB concentration in conditioned media from NC/Id2‐siRNA‐transfected BMMs receiving different treatments were examined by ELISA. n = 3 per group. *P < .05, **P < .01, ***P < .001

We next investigated the involvement of Id2 in the harmine‐induced promotion of preosteoclast formation and PDGF‐BB production. As evidenced by qRT‐PCR analysis, RANKL‐induced BMMs expressed lower Id2 mRNA levels compared to the un‐induced group, while harmine treatment significantly increased the Id2 mRNA levels in BMMs (Figure 2A). Western blotting further confirmed the stimulatory effect of harmine on Id2 protein expression (Figure 2B). To verify the role of Id2 in the differentiation of osteoclast, special siRNAs were transfected into BMMs to down‐regulate the expression of Id2. The inhibitory efficiency of Id2 siRNA (Id2‐siRNA) was confirmed by qRT‐PCR for Id2 gene (Figure 2C) and Western blotting analysis for Id2 protein (Figure 2D). TRAP staining showed that Id2 suppression markedly down‐regulated the ability of harmine to inhibit the fusion of mononuclear preosteoclasts into multinucleated osteoclasts, as revealed by the significantly increased number of osteoclasts and reduced number of preosteoclasts in Id2‐siRNA + RANKL + harmine group compared with NC‐siRNA + RANKL + harmine group (Figure 2E‐G). Accompanying with the decrease of preosteoclasts, Id2‐down‐regulated BMMs showed lower expression of PDGF‐BB at both mRNA and protein levels compared with control BMMs after stimulation with harmine (Figure 2H and I). However, in BMMs treated with vehicle or RANKL only, inhibition of Id2 did not notably affect the formation of osteoclasts, preosteoclasts and PDGF‐BB production (Figure 2E‐I). These results suggest that Id2 plays a positive role in harmine‐increased preosteoclast formation and PDGF‐BB production.

### 3.3 AP‐1 is essential for the positive effect of harmine on preosteoclast PDGF‐BB production

**FIGURE 3:**
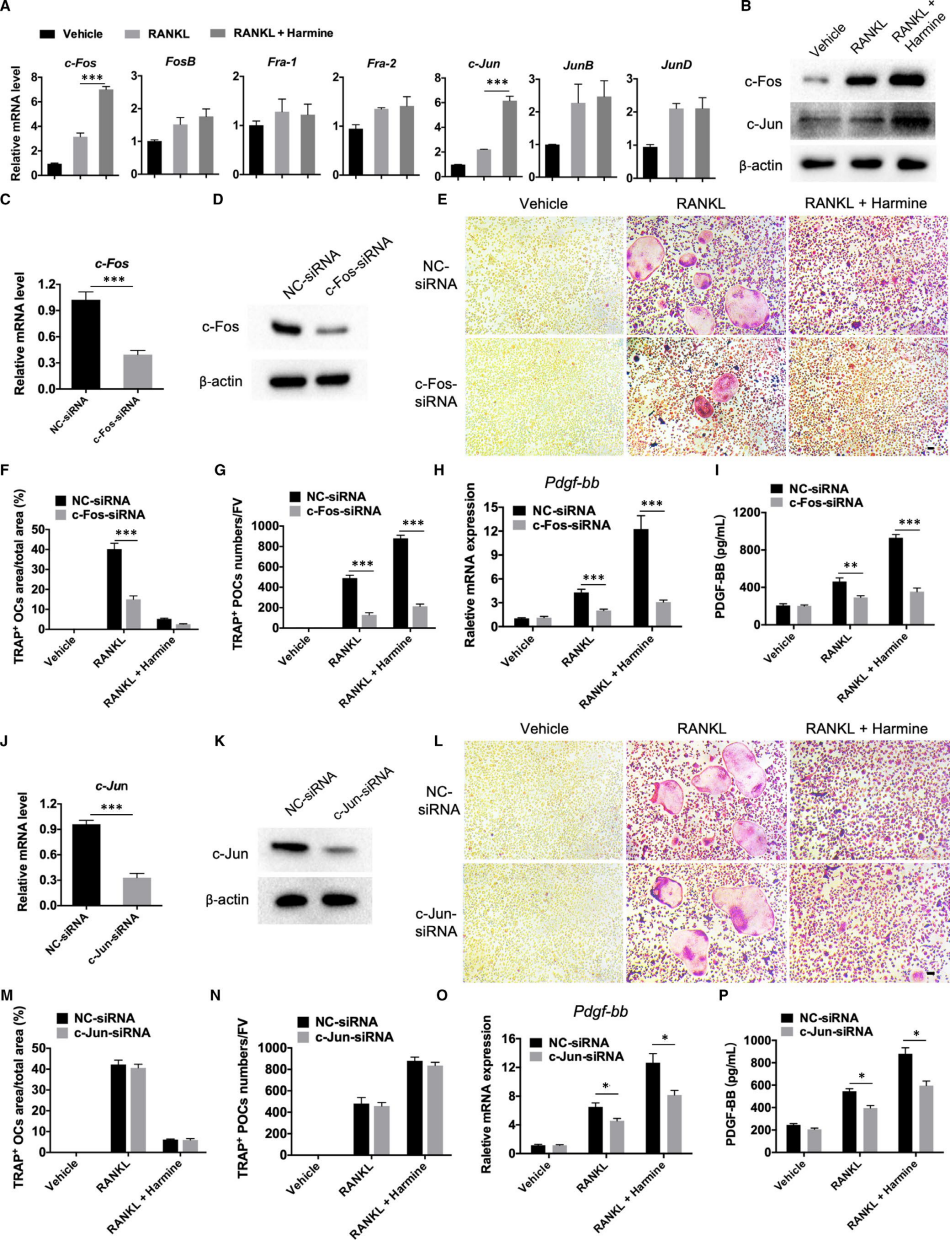
AP‐1 is essential for the positive effect of harmine on preosteoclast PDGF‐BB production. A, qRT‐PCR analysis of the expression levels of AP‐1‐related components including c*‐Fos, FosB, Fra‐1, Fra‐2, c‐Jun, JunB* and *JunD* in BMMs treated with vehicle, RANKL, RANKL + harmine. n = 3 per group. B, Western blot analysis of the protein levels of c‐Fos and c‐Jun in BMMs receiving different treatments. C, D, Transfection of BMMs with c‐Fos‐siRNA led to a decrease in c‐Fos expression levels as verified by qRT‐PCR C, and Western blotting analysis D, n = 3 per group. E‐G, Representative TRAP staining images and quantitative analyses of osteoclasts F, and preosteoclasts G, from NC/c‐Fos‐siRNA‐transfected BMMs treated with vehicle, RANKL, or RANKL + harmine. Scale bar = 50 μm. n = 3 per group. H, qRT‐PCR analysis of PDGF‐BB mRNA expression levels in groups receiving different treatments as indicated. n = 3 per group. I, PDGF‐BB concentration in conditioned media from BMMs in different groups was detected by ELISA. n = 3 per group. J, K, BMMs transfected with c‐Jun‐siRNA showed a decrease in c‐Jun expression levels as determined by qRT‐PCR J, and Western blotting analysis K, n = 3 per group. L‐N, Representative TRAP staining images L, and quantitative analyses of osteoclasts M, and preosteoclasts N, from NC/c‐Jun‐siRNA‐transfected BMMs receiving different treatments. Scale bar = 50 μm. n = 3 per group. O, qRT‐PCR analysis of PDGF‐BB mRNA expression levels in different groups. n = 3 per group. P, PDGF‐BB concentration in conditioned media from different groups was detected by ELISA. n = 3 per group. *P < .05, **P < .01, ***P < .001

As Id2 down‐regulation only partially blocked the stimulatory effect of harmine on preosteoclast PDGF‐BB production, we then examined whether other molecular mechanism is involved in this process. We analysed the promoter region of the PDGF‐BB gene and investigated the potential PDGF‐BB‐related transcription factors. The results revealed that the promoter region of PDGF‐BB contained a unique putative binding site for AP‐1. Thus, we examined the expression of the components of AP‐1 in each group. qRT‐PCR analyses determined that RANKL treatment profoundly increased the mRNA levels of a lot of AP‐1 factors in BMMs, whereas only the expression of *c‐Fos* and *c‐Jun* was further enhanced in response to harmine co‐incubation (Figure 3A). Western blotting confirmed the augmented protein levels of c‐Fos and c‐Jun in RANKL + harmine‐treated BMMs compared with BMMs treated with vehicle or RANKL only (Figure 3B), suggesting that these two factors might be involved in the harmine‐induced preosteoclast PDGF‐BB production. To confirm our hypotheses, we constructed the specific targeting siRNAs to respectively knockdown *c‐Fos* and *c‐Jun* in BMMs. Knockdown of *c‐Fos* in BMMs was confirmed by qRT‐PCR (Figure 3C) and Western blotting (Figure 3D). Down‐regulation of c‐Fos not only markedly blocked the RANKL‐induced formation of osteoclasts and preosteoclasts and the expression of PDGF‐BB at the gene and protein levels, but also remarkably inhibited the harmine‐induced promotion of preosteoclast formation and PDGF‐BB production, as revealed by TRAP staining (Figure 3E‐G), qRT‐PCR for *Pdgf‐bb* gene (Figure 3H) and ELISA for PDGF‐BB protein in the conditioned media (Figure 3I). However, knockdown of *c‐Jun* in BMMs (Figure 3J and K) did not induce obvious effects on osteoclast and preosteoclast formation (Figure 3L‐N), but notably blocked PDGF‐BB expression at the gene and protein levels in both RANKL‐ and RANKL + harmine‐treated BMMs (Figure 3O‐P), suggesting a direct effect of harmine on PDGF‐BB expression by targeting AP‐1.

### 3.4 AP‐1 directly targets PDGF‐BB

**FIGURE 4:**
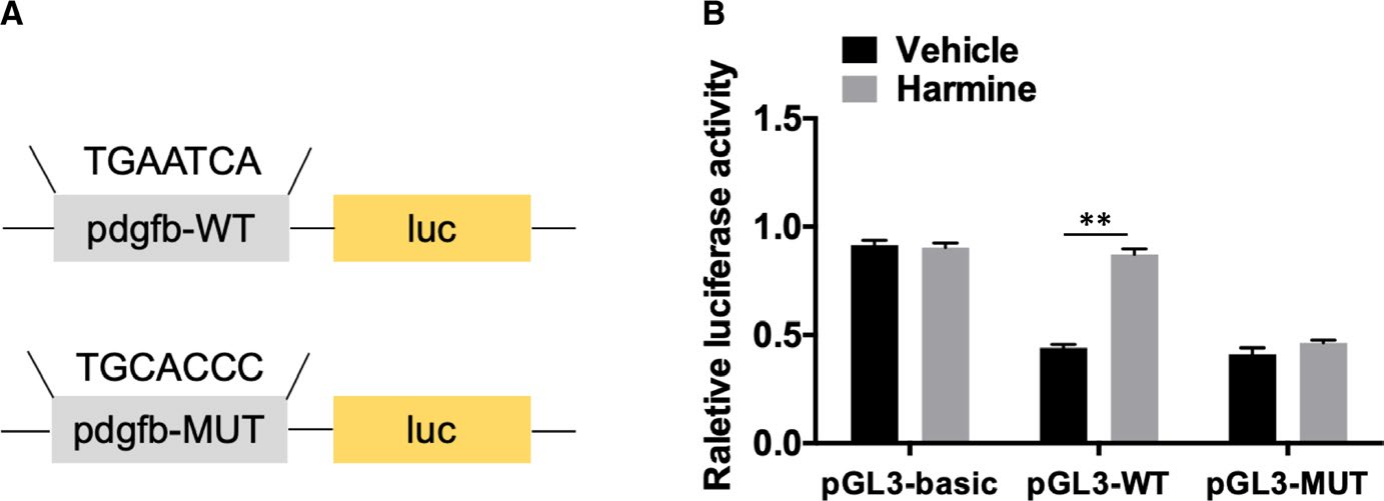
AP‐1 directly targets PDGF‐BB. A‐B, A luciferase reporter plasmid containing wild‐type or mutant binding site for AP‐1 was transfected into BMMs treated with vehicle or harmine. The luciferase activity was assessed by Dual‐Luciferase reporter assay system. *P < .05, **P < .01, ***P < .001

To further explore whether PDGF‐BB is a direct target of AP‐1, we constructed luciferase reporter plasmid driven by PDGF‐BB promoter region containing the putative binding site for AP‐1. Mutant constructs were generated using site‐specific mutagenesis (Figure 4A). Dual‐luciferase reporter assay demonstrated that in BMMs transfected with WT constructs, harmine treatment significantly augmented luciferase activity compared to vehicle treatment group; however, harmine did not affect the luciferase activity in cells transfected with the MUT constructs (Figure 4B), which confirmed the direct action of harmine on PDGF‐BB expression through AP‐1‐mediated transcriptional activation. Nuclear translocation analyses also confirmed a much higher levels of AP‐1 in the nuclei of RANKL‐induced BMMs treated with harmine relative to that of vehicle group (Figure S3).

## 4 DISCUSSION

Osteoporosis is systemic skeletal disorder characterized by elevated bone resorption and increased risk of fracture. Strategies for fracture prevention mainly include antiresorptive therapy and anabolic therapy. Bisphosphonates, widely used as first‐line drugs for the treatment of osteoporosis, are highly effective in suppressing bone resorption and reducing fracture risk via inhibiting the recruitment and activity of osteoclasts.^17^ However, it has been described that long‐term use of bisphosphonates is associated with several side effects (such as osteonecrosis of the jaw, atypical femoral fracture).^18^, ^19^ Harmine is a natural small‐molecule alkaloid. Accumulating evidence indicates that harmine exerts positive effects on bone metabolism, which makes harmine a novel candidate for the treatment of bone loss diseases.^13^, ^14^, ^20^ Our previous study also found that harmine prevents bone loss in ovariectomized mice via promoting PDGF‐BB secretion and enhancing type H vessel formation.^6^ In the present study, we further revealed the underlying molecular mechanism that harmine stimulates PDGF‐BB generation by promoting Id2 and AP‐1 expression during the process of osteoclastic differentiation. However, the administration of harmine may cause several adverse effects, such as neurotoxicity.^21^, ^22^ The lack of clinical data is also a challenge. It still has a long way to go before the clinical application of harmine as a therapeutic drug for osteoporosis.

Osteoclasts are differentiated from monocyte/macrophage lineage, which undergoes sequential stages including preosteoclast formation and cell‐cell fusion, involving a variety of proteins and kinases.^23^, ^24^, ^25^ Id2 is a member of Id family, which functioned as inhibitor of cell differentiation.^26^ Consistent with previous data,^13^ we found that Id2, up‐regulated by harmine, suppressed preosteoclasts to fuse into multinucleated osteoclasts, and Id2 knockdown reversed the effects of harmine. Given that preosteoclasts, not osteoclasts, mainly secret PDGF‐BB,^8^ our results indicated that harmine boosted PDGF‐BB production based on increasing the quantity of preosteoclasts, which was mediated by Id2. Egusa et al ^13^ showed that harmine also affected the expression of DC‐STAMP which is essential for cell‐cell fusion in osteoclasts.^27^ However, we cannot exclude the possibility that other mechanism of harmine‐mediated increase in preosteoclast formation may exist, which warrants further investigation.

AP‐1, a dimeric transcription factor composed of Fos and Jun proteins, is a key positive regulator in osteoclast differentiation initially activated by RANKL signal.^28^ In this study, we found that harmine significantly augmented AP‐1 factors (c‐Fos and c‐Jun) expression in RANKL‐induced BMMs, and c‐Fos played a positive role in harmine‐induced preosteoclast formation. Knockdown of c‐Fos or c‐Jun both reduced PDGF‐BB secretion stimulated by harmine. Further examination revealed that AP‐1 directly activated PDGF‐BB transcription. Thus, in addition to enhancing preosteoclast formation (combining with the fusion‐inhibition effect of Id2), AP‐1 is also able to directly promote PDGF‐BB expression, indicating a dual functional role of AP‐1 during the process of preosteoclast PDGF‐BB production. In this study, we also observed that c‐Jun knockdown presented no obvious impact on RANKL‐induced osteoclast differentiation. The result is likely attributed to the compensating effects of other Jun members for osteoclast formation.^29^ Taken together, our findings suggest that the harmine‐induced increase of PDGF‐BB generation during osteoclastogenesis is not only contributed to its positive role in preosteoclast formation, but also owing to its direct stimulatory action on PDGF‐BB expression through the up‐regulation of AP‐1 transcription factors.

However, these molecular mechanism studies were only done in vitro, but not in vivo. We expect to verify the results in gene knockout mice in future if conditions permit.

In summary, our study provides mechanistic insight into how harmine regulates PDGF‐BB production during osteoclast differentiation, which could enlighten novel therapeutic target for prevention and treatment of bone metabolic diseases.

## 5 CONCLUSION

In the present study, we found that harmine stimulates PDGF‐BB production via promoting the expression of Id2 and AP‐1 during the process of osteoclastic differentiation. Our work confers a promising therapeutic target for bone loss diseases.

## CONFLICT OF INTEREST

The authors confirm that there are no conflicts of interest.

## AUTHOR CONTRIBUTIONS

**Jie Huang:** Formal analysis (lead); Investigation (lead); Methodology (lead). **You‐You Li:** Investigation (lead); Methodology (equal); Project administration (equal); Resources (equal). **Kun Xia:** Investigation (equal); Methodology (equal). **Yi‐Yi Wang:** Formal analysis (equal); Investigation (equal); Methodology (equal). **Chun‐Yuan Chen:** Methodology (equal). **Meng‐Lu Chen:** Methodology (equal). **Jia Cao:** Methodology (equal). **Zheng‐Zhao Liu:** Methodology (equal). **Zhen‐Xing Wang:** Methodology (equal). **Hao Yin:** Methodology (equal). **Xiong‐Ke Hu:** Methodology (equal). **Zheng‐Guang Wang:** Resources (equal); Visualization (equal). **Yong Zhou:** Supervision (equal); Writing‐review & editing (equal). **Hui Xie:** Funding acquisition (lead); Project administration (lead); Visualization (lead); Writing‐original draft (lead); Writing‐review & editing (lead).

## Supporting information

Supplementary Material
