# Supplementary material for: Harmine targets inhibitor of DNA binding‐2 and activator protein‐1 to promote preosteoclast PDGF‐BB production
Source: J Cell Mol Med. 2021 May 7;25(12):5525–33. doi: 10.1111/jcmm.16562 (PMC8184727; doi:10.1111/jcmm.16562)
Supplement: Supplementary file 1 — Supplementary Material [file JCMM-25-5525-s001.docx]

**Supplemental material and methods**

**Harmine targets inhibitor of DNA binding-2 (Id2) and activator protein-1 (AP-1) to promote preosteoclast platelet-derived growth factor-BB (PDGF-BB) production**

Jie Huang^1,2#^, You-You Li^1,2#^, Kun Xia^1,2^, Yi-Yi Wang^1,2^, Chun-Yuan Chen^1,2^, Meng-Lu Chen^1,3^, Jia Cao^1,2^, Zheng-Zhao Liu^1,3,6^, Zhen-Xing Wang^1,2^, Hao Yin^1,2^, Xiong-Ke Hu^1,2^, Zheng-Guang Wang^7^*, Yong Zhou^7^* and Hui Xie^1-6^*

1. Department of Orthopedics, Xiangya Hospital, Central South University, Changsha, Hunan 410008, China.
2. Movement System Injury and Repair Research Center, Xiangya Hospital, Central South University, Changsha, Hunan 410008, China.
3. Department of Sports Medicine, Xiangya Hospital, Central South University, Changsha, Hunan 410008, China.
4. Hunan Key Laboratory of Organ Injury, Aging and Regenerative Medicine, Changsha, Hunan 410008, China.
5. Hunan Key Laboratory of Bone Joint Degeneration and Injury, Changsha, Hunan 410008, China.
6. National Clinical Research Center for Geriatric Disorders, Xiangya Hospital, Central South University, Changsha, Hunan 410008, China.
7. Department of Orthopedics Third Xiangya Hospital, Central South University, Changsha, Hunan 410013, China.

^#^ Jie Huang and You-You Li contributed equally to this work.

* Corresponding authors:

Hui Xie, Ph.D., Department of Orthopedics, Xiangya Hospital, Central South University, #87 Xiangya Road, Changsha, Hunan 410008, China. E-mail: [huixie@csu.edu.cn](mailto:huixie@csu.edu.cn).

Yong Zhou, M.D., Department of Orthopedics, Third Xiangya Hospital, Central South University, #138 Tongzipo Road, Changsha, Hunan 410013, China. E-mail: [zhouyong1028@sina.com](mailto:zhouyong1028@sina.com).

Zheng-Guang Wang, M.D., Department of Orthopedics, Third Xiangya Hospital, Central South University, #138 Tongzipo Road, Changsha, Hunan 410013, China. E-mail: [wzg19830216@163.com](mailto:wzg19830216@163.com).

**Supplemental Table 1. Primers for Real time qPCR.**

| **Name** | **Sequence** |
| --- | --- |
| mouse Id2 forward | 5'-ATGAAAGCCTTCAGTCCGGTG-3' |
| mouse Id2 reverse | 5'-GGTTCAAGGTCATGCTCTGTTT-3' |
| mouse c-Fos forward | 5'-CGGGTTTCAACGCCGACTA-3' |
| mouse c-Fos reverse | 5'-TTGGCACTAGAGACGGACAGA-3' |
| mouse FosB forward | 5'-TTTTCCCGGAGACTACGACTC-3' |
| mouse FosB reverse | 5'-GTGATTGCGGTGACCGTTG-3' |
| mouse Fra-1 forward | 5'-ATGTACCGAGACTACGGGGAA-3' |
| mouse Fra-1 reverse | 5'- CTGCTGCTGTCGATGCTTG-3' |
| mouse Fra-2 forward | 5'-CCAGCAGAAGTTCCGGGTAG-3' |
| mouse Fra-2 reverse | 5'-GTAGGGATGTGAGCGTGGATA-3' |
| mouse c-Jun forward | 5'-CCTTCTACGACGATGCCCTC-3' |
| mouse c-Jun reverse | 5'- GGTTCAAGGTCATGCTCTGTTT-3' |
| mouse JunB forward | 5'-TCACGACGACTCTTACGCAG-3' |
| mouse JunB reverse | 5'-CCTTGAGACCCCGATAGGGA-3' |
| mouse JunD forward | 5'-GAAACGCCCTTCTATGGCGA -3' |
| mouse JunD reverse | 5'-CAGCGCGTCTTTCTTCAGC-3' |
| mouse Pdgf-bb forward | 5'-CCTCGGCCTGTGACTAGAAG-3' |
| mouse Pdgf-bb reverse | 5'-CCTTGTCATGGGTGTGCTTA-3' |
| mouse Gapdh forward | 5'-CACCATGGAGAAGGCCGGGG-3' |
| mouse Gapdh reverse | 5'-GACGGACACATTGGGGGTAG-3' |

**Supplemental figure 1. Harmine increases the number of preosteoclasts, the production of bone marrow PDGF-BB and type H vessel formation in ovariectomized (OVX) mice.** (A) TRAP staining of femora from sham, OVX and OVX + harmine mice. Black arrows indicate preosteoclasts. Scale bar: 50 μm. (B) Quantification of TRAP+ osteoclasts (OCs) and preosteoclasts (POCs) per bone surface (BS) in different treatment groups. n = 4 per group. (C) Bone marrow concentrations of PDGF-BB from different treatment groups. n = 4 per group. (D) Representative images of CD31 (green) and Emcn (red) immunostaining in femora from sham, OVX and OVX + harmine mice. BM: bone marrow; GP: growth plate; TB: trabecular bone. Scale bar: 100 μm.**P* < 0.05.


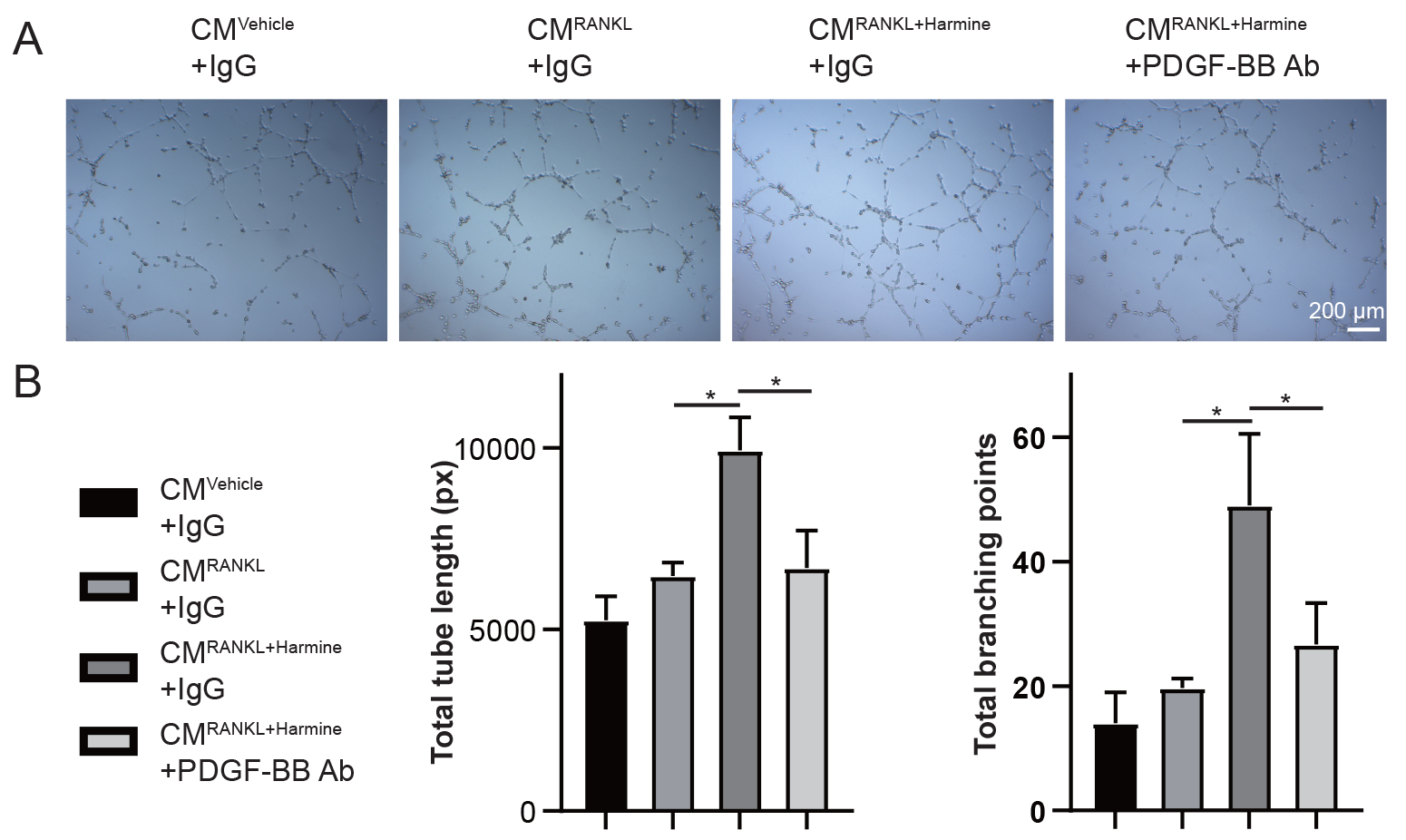


**Supplemental figure 2. Harmine augments the pro-angiogenic effects of preosteoclasts via enhancing PDGF-BB production.** (A-B) Representative images and quantification of tube formation in Human microvascular endothelial cells stimulated with conditioned media (CM) from different groups and PDGF-BB-neutralizing antibody or IgG isotype control antibody. Scale bar: 200 μm. n = 3 per group. **P* < 0.05.


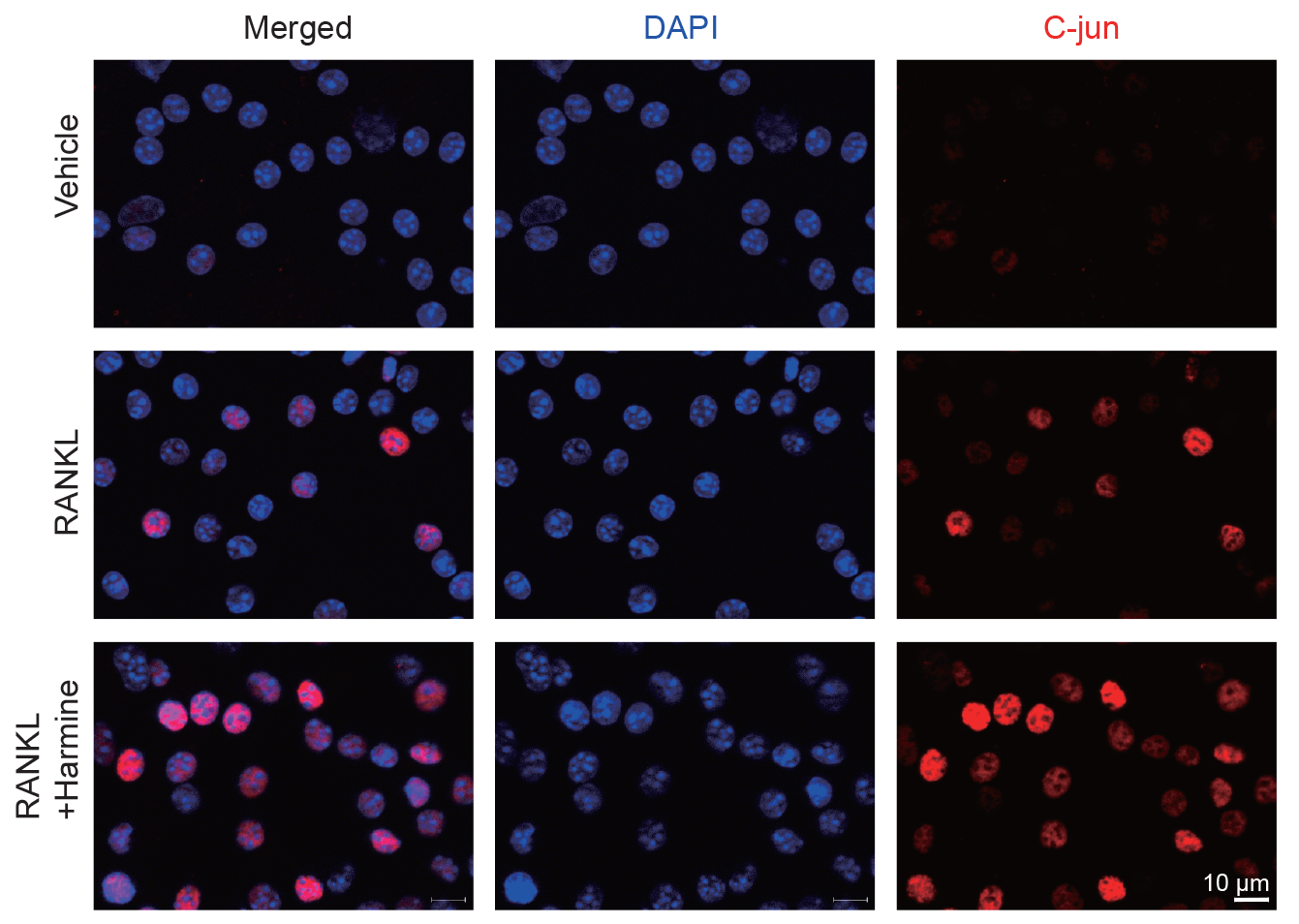


**Supplemental figure 3. Harmine promotes AP-1 nuclear translocation in RANKL-induced bone marrow macrophages (BMMs).** Representative images of AP-1 (red) immunostaining RANKL-induced in BMMs treated with or without harmine. Nuclei were stained with DAPI. Scale bar: 10 μm.

**Methods**

**Animals and treatments**

To evaluate the effect of harmine on preosteoclast PDGF-BB production *in vivo*, 12-week-old female C57BL/6 mice were used and randomly divided into three groups: (1) Sham group: mice subjected to sham operation and treated with vehicle; (2) OVX group: mice subjected to ovariectomy and treated with vehicle; (3) Harmine group: mice subjected to ovariectomy and intragastrically treated with harmine solution (10 mg/kg/day). After four weeks’ administration, the mice were euthanized. The femur samples and bone marrow from tibias of mice were harvested for further examinations. Animal experiments were reviewed and approved by the Animal Research Committee of Central South University.

**Tartrate-resistant acid phosphatase (TRAP) staining**

The femora were fixed in 4% paraformaldehyde for 24 h, decalcified in 18% ethylene diamine tetra acetic acid (EDTA) for 3 days. Then, samples were embedded in paraffin and cut into 5‐μm thick sections. Trap staining was performed with Trap stain kit (sigma) according to the instructions provided by the manufacturer. The number of preosteoclasts or osteoclasts per adjacent bone surface (N/mm) were calculated. TRAP positive (red) mononuclear cells and multinucleated (three and more nuclei) cells were counted as preosteoclast and osteoclast, respectively.

**Enzyme linked immunosorbent assay (ELISA)**

The bone marrow was harvested and stored at –80°C until analysis. The concentration of PDGF-BB was detected with commercial ELISA kit (Elabscience, China) following the instructions provided by the manufacturer.

**Tube formation assay**

Human microvascular endothelial cells (HMECs) were cultured in MCDB131 medium (Gibco) containing 10% FBS (Gibco), 1 μg/mL hydrocortisone (Sigma, St. Louis, MO, USA), 2 mM L-glutamine (Sigma) and 10 ng/mL epidermal growth factor (EGF; Sigma). Then, HMECs were seeded in the 96-well plate (1×10^4^ per well) paved with matrigel (Corning Matrigel Matrix GFR) and treated with different conditioned media (supernatant from un-induced, RANKL-treated or RANKL + harmine-treated BMMs) and PDGF-BB-neutralizing antibody (Abcam) or IgG isotype control antibody (Abcam). After incubation at 37℃ for 6 h, tube formation was observed and photographed by inverted microscope (Leica). Total tube length, and total branching points was measured by Image Pro Plus 6.0 software.

**Immunofluorescent analyses**

Femur Samples were fixed by 4% paraformaldehyde for 24 h and decalcified in 18% EDTA solution for 3 days. Then the samples were dehydrated by 30% sucrose and embedded by OCT (Opti-mum Cutting Temperature Compound) for freeze sections. After washed with PBS for three times, sections were incubated with primary antibody (CD31, 1:50, Abcam; EMCN, 1:100, Santa Cruz ) overnight at 4℃. After that, sections were incubated with secondary antibody at room temperature for 1 h. Nuclei was stained by DAPI. Images were observed and analyzed by Axio Imager 2 Apotome (ZEISS) fluorescence microscope. For AP-1 nuclear translocation analysis, primary BMMs were seeded in 24-well plate coverslips (Solarbio) and treated with 100ng/mL RANKL, 30ng/mL M-CSF and 3μM Harmine or equal volume solvent (DMSO). After 4 days of induction, cells were washed with PBS, fixed by 4% paraformaldehyde for 15min, and incubated with primary antibody (c-Jun, 1:300, CST) overnight at 4℃ (As AP-1 is a dimeric molecule, we detected c-jun, a component of AP-1, to determine the expression of AP-1). Coverslips were exposed with Cy3-conjugated secondary antibody at room temperature for 1h. After washed with PBS for three times, nucleus was stained by DAPI. Images were captured by Axio Imager 2 Apotome (ZEISS) fluorescence microscope.
